# Evaluating the impact of “Sustainable Urban Mobility Plans” on urban background air quality

**DOI:** 10.1016/j.jenvman.2018.10.039

**Published:** 2019-02-01

**Authors:** E. Pisoni, P. Christidis, P. Thunis, M. Trombetti

**Affiliations:** a European Commission, Joint Research Centre (JRC), Directorate for Energy, Transport and Climate, Air and Climate Unit, Via E. Fermi 2749, I-21027, Ispra, VA, Italy; b European Commission, Joint Research Centre (JRC), Directorate for Energy, Transport and Climate, Economics of Climate Change, Energy and Transport, Edificio Expo, C/ Inca Garcilaso, 3, 41092, Sevilla, Spain; c European Topic Centre on Urban, Land and Soil Systems (ETC/ULS), University of Malaga, C/Arquitecto Francisco Penalosa, 18, 29071, Malaga, Spain

**Keywords:** Sustainable urban mobility plans, Low emission zones, Transport modelling, Air quality modelling

## Abstract

Air quality in European cities is still a challenge, with various urban areas frequently exceeding the PM_2.5_ and NO_2_ concentration levels allowed by the European Union Air Quality Standards. This is a problem both in terms of legislation compliance, but also in terms of health of citizens, as it has been recently estimated that 400 to 450 thousand people die prematurely every year due to poor air quality. Air quality in cities can be improved with a number of interventions, at different sectoral (industry, traffic, residential, etc …) and geographical (international, European, national, local, etc.) levels. In this paper we explore the potential of city level plans to improve mobility and air quality (excluding electro-mobility options, not considered in this study). We applied the “Sustainable Urban Mobility Plans” (SUMPs) framework to 642 cities in Europe and modelled how the measures they include may impact at first on mobility and emissions at urban level, and then on urban background concentrations of PM2.5 and NO2. Results show that annual averages moderately improve for both pollutants, with reductions of urban background concentrations up to 2% for PM_2.5_ and close to 4% for NO_2_. The impact on NO_2_ at street level (that will be higher than on urban background) is not evaluated in this work. The air quality improvement of the simulated SUMP would only partially alleviate air quality problems in urban areas, but such a reduction in the emissions of air pollutants should still be considered as a positive result of SUMPs, given that they correspond to a set of low-cost measures that can be implemented at local level. Furthermore, the introduction of electro-mobility options (not considered here) would increase the impact on air quality. Other types of benefits, such as reduced fuel consumption, greenhouse gas emissions, higher impact at street level or accident rates reduction further add to the overall positive impact.

## Highlights

• SUMPs (Sustainable Urban Mobility Plans) concept have been applied to 642 cities In Europe.
• Impact on modelled “urban background concentrations” has been evaluated.
• Results show that this impact is limited for “urban background concentrations”.
• The impact of SUMPs on street level concentrations, and electro-mobility options, are not evaluated in this paper.
• Other benefits of SUMPs (on greenhouse gases, reduced accident rates, etc …) should be also considered.

## 1 Introduction

Many city dwellers are exposed to high level of air pollution. It has been estimated (EEA, 2017) that more than 80–82% of urban population in Europe are exposed to PM_2.5_ concentrations above WHO guidelines. At the same time, the annual limit value for nitrogen dioxide concentrations (NO_2_) continues to be widely exceeded across Europe, with around 10% of all reporting stations recording concentrations above the Air Quality Standard threshold values (in 2015) in a total of 22 of the EU-28 states (89% of all concentration exceedances were observed at traffic stations). This situation calls for action, and this is happening at various decision levels. The recent review of the Gothenburg Protocol and the new National Emission Ceiling Directive (EU, 2016) both go in this direction, approaching the problem from an international perspective. Local authorities can also play a role, and there are multiple efforts also at local scale to evaluate pollution concentrations (Carnevale et al., 2011) and the impact of local policies on air quality (Carnevale et al., 2014).

In this work we focus on “Sustainable Urban Mobility Plans”, a concept/framework developed to support local level authorities in exploring new urban mobility strategies. From the point of view of urban mobility, various options are available, including congestion charges, car sharing schemes, promotion of eco-driving, etc … There are now numerous examples of applications of such measures in European cities; for a summary of applications one can look at the paper of Holman et al. (2015), in which the authors performed a review e.g. of the efficacy of low emission zones in improving air quality in European cities. The authors analysed the results from 5 European countries (Denmark, Germany, Netherlands, Italy and UK), in which low emission zones where applied in different configurations (i.e. only for Buses, Heavy goods vehicles or passenger cars, etc …). While for German cities annual mean PM_10_ and NO_2_ was up to 7% and 4% lower, no effects were observed on air quality for the other cases. The authors also suggested that one should look at carbonaceous particles (not PM or NO_2_) to evaluate the impact of low emission zones, as these are more affected by traffic and consequently the impact of low emission zones is more pronounced.

It is also true that the impact of congestion/charges low emission zones depend on how the measure is implemented. I.e. Ferreira et al., 2015, showed how the implementation of a low emission zone in Lisbon could give a reduction in PM_10_ annual average concentrations of 23%, and NO_2_ of 12%. However the implementation of the measure was assumed to be ‘sharper’ than in other cases.

Looking at the problem more from a health perspective, Cyrys et al., 2014 stated that in Germany there were (in 2014) 45 low emission zones, for which it has been estimated (by means of dispersion modelling, but also through analysis of concentrations levels after the low emission zones implementation) that PM_10_ should decrease by up to 10%. At the same time, the authors showed that diesel soot (that is particularly toxic) to be preferentially targeted by these measures; as in for example the case of Berlin, where concentrations of traffic-related soot along major roads decreased by more than 50%. The authors therefore stated that LEZ could be more beneficial for human health than the impression one would have from only looking at the change in concentrations of the regulated pollutants. Health has been also the focus of another study (Cesaroni et al., 2012), aiming to evaluate the impact, in terms of air quality and health effects, of two low-emission zones established in Rome in the period 2001–2005 and to assess the impact by socioeconomic position of the population.

In the case of London, Ellison et al., 2013, showed that one impact of the low emission zones was to enhance fleet turnover (the low emission zone implementation ‘cleaned’ the circulating fleet). But in terms of impact of concentrations, they found values similar to Holman et al., with particulate matter falling by roughly 3% in the low emission zone, and no discernible impact on NO_x_ concentrations.

From a broader perspective, Bigazzi et al., 2017 reviewed the effectiveness of traffic management strategies for improving air quality. After having performed a systematic literature search to identify empirical studies, they found that only 7 of 22 studied strategies had an impact on emission reductions, and 2 of the strategies had a limited impact on air quality: area road pricing and low emission zones. To reach a significant air quality impact, the authors state it is important to implement ‘aggressive’ measures, but also to consider possible unintended detrimental and rebound effects (as i.e. the increase of emissions in areas surrounding the low emission zones).

Also ex-ante evaluation studies exist, as in the case of Dias et al. (2016), in which an integrated modelling approach was proposed to evaluate the impact of LEZ. The proposed model was linking a transportation model with an emissions model and an air quality model operating over a GIS-based platform, allowing for estimating the changes induced by the creation of a LEZ not only inside the LEZ, but also, more generally, in the city where it is located.

Starting from this brief literature overview, we defined our main policy research. The main aim of our work is to evaluate how mobility measures can impact air quality at city scale, focusing on NO2 and PM2.5 yearly average improvement. In particular, in this paper we followed a similar approach to Bigazzi et al., 2017, starting from a possible list of interventions that cities can implement from the mobility point of view. As SUMP (Sustainable Urban Mobility Plans) provide a framework for mobility strategies at urban level, this has been considered as a reference. Starting from this idea, and for the first time at the knowledge of the authors, an attempt has been implemented to link, through a modelling chain.

- the design of SUMP for different city types, encompassing different packages of measures depending on the city features (without considering electro-mobility options);
- their impact on emission at urban level;
- their impact on urban background concentrations.

A list of the considered cities can be found in the “supplementary information”. A caveat of the work is that urban policies will impact both urban background and street level concentrations; however, this paper only focus on the urban background impact of such policies. The structure of the paper is as follows: in Section 2 the modelling chain used is presented, in Section 3 the resulting measure package, emissions and concentrations, and in Section 4 a short discussion and the conclusions.

## 2 Methodology

The innovative aspect of the methodology we present here is the integration of two different modelling approaches in order to assess the impact of SUMPs at urban level. The approach consists of two steps:

• Simulation of the impacts of the various measures on mobility depending on the urban profile in order to estimate potential changes in emissions from transport (using the T-NET/TRANSTOOLS models).
• Decomposition of urban emissions by source and the evaluation of new levels of air quality assuming the simulated changes in emissions from transport (using the SHERPA model).

### 2.1 T-NET/TRANSTOOLS

The T-NET/TRANSTOOLS model family (Ibañez et al., 2016; Christidis and Ibañez, 2012) is a set of European transport network models covering both passengers and freight, as well as intermodal transport. It combines advanced modelling techniques in transport generation and assignment, economic activity, trade, logistics, regional development and environmental impacts.

Among other applications, T-NET/TRANSTOOLS has been used as part of the Impact Assessment of “Sustainable Urban Mobility Plans” (http://www.eltis.org/mobility-plans/sump-concept) to explore the environmental impacts of different urban mobility plans. In particular, the analysis estimated the impact that a mix of policy measures would have on urban-level CO2 emissions for each NUTS-3 zone in Europe.^1^ Depending on the characteristics of each NUTS-3 zone (see Lopez-Ruiz et al., 2013) a different mix of sustainable mobility measures was assumed with varying levels of corresponding emissions reductions.

The original analysis is extended here through two major improvements that allow more detailed input to be provided to the SHERPA model:

• Refinement of the spatial level of the analysis: the zoning system of the transport model was further refined to sub-NUTS-3 levels (i.e. zones smaller than a province) by distinguishing urban areas within each NUTS-3 zone. For each NUTS-3 zone the Functional Urban Areas (FUA) according to the EU-OECD definition^2^ were identified and their specific transport activity was estimated. Such a distinction of urban and non-urban transport activity within each NUTS-3 zone allows greater precision in the allocation of activity and emissions and ensures that the spatial distribution of emissions that will be taken into account by SHERPA will be closer to reality.
• Extension of analysis of impacts to NO_x_ emissions: while the original focus of the Impact Assessment of SUMPS was climate change, the main interest from the SHERPA point of view lies with air quality pollutants. As a consequence, new emission factors for NO_x_ from COPERT 5 (Gkatzoflias et al., 2012) were introduced into T-NET. The impact of SUMPs was re-calculated assuming that all FUAs in a Member State have the same technological and fuel mix that T-NET/TRANSTOOLS simulate endogenously (iTREN-2030 project, 2009) since such data is only available at national level.

These two main improvements allowed the results of the analysis to show higher variability within each Member State (since the specific FUA characteristics vary more than the ones at NUTS-3 level and NUTS-3 zone may contain more than two urban and non-urban parts) and a better match between the NO_x_ levels estimated by T-NET/TRANSTOOLS and the NO_2_ concentrations calculated by SHERPA. The decomposition of the results also allows the different groups of Sustainable Urban Mobility measures to be ranked in terms of their contribution to the reduction of emissions.

### 2.2 SHERPA

SHERPA (Clappier et al., 2015; Thunis et al., 2016; Pisoni et al., 2017, 2018) has been developed to provide a fast modelling approach to calculate concentration fields resulting from emission reduction scenarios, which mimics the behavior of a full Chemical Transport Model (CTM). CTMs (Candiani et al., 2013; Pernigotti el al., 2013) provide pollutant concentration fields that account for the complex transport, diffusion and chemical processes happening in the atmosphere. The aim of SHERPA is to mimic a CTMs' behavior using a simple relation derived from a set of full CTM simulations built with various emission reduction scenarios.

In SHERPA, concentration changes due to an emission reduction scenario are computed on a cell by cell basis according to the following equation:

(1) $${\text{ΔC}}_{\text{n}}=\sum_{\text{p}}^{{\text{N}}_{\text{prec}}}\sum_{\text{m}}^{{\text{N}}_{\text{cell}}}{\text{a}}_{\text{n,p,m}}{\text{ΔE}}_{\text{p,m}},\forall n\in \left[1,N_{cell}\right],$$

where the delta concentration ΔC_n_ (change of concentration in comparison to the base case) in a receptor grid cell “n” is expressed as a linear combination of the emissions delta $\Delta {\text{E}}_{\text{p,m}}$ (variation in emission when compared to the base case), for each source cell “m” and pollutant (i.e. precursor) “p”. The ${\text{a}}_{\text{n,p,m}}$ coefficients act as weighting factors which apportion the amount of emission variation $\Delta {\text{E}}_{\text{p,m}}$ of precursor p stemming from cell m and reaching cell n. As the correlation between ${\text{ΔC}}_{\text{n}}$ (at receptor cell n) and ${\text{ΔE}}_{\text{p,m}}$ (at all sources cell m) decreases with the distance between the cells, it has been assumed that the coefficients ${\text{a}}_{\text{n,p,m}}$ in the previous equation can be approximated by the following distance-function:

(2) $${\text{a}}_{\text{n,p,m}}={\text{α}}_{\text{n,p}}{\left(1+{\text{d}}_{\text{n,m}}\right)}^{-{\text{ω}}^{\text{n,p}}}$$

where d_n,m_ is the distance between cells n and m and the two unknowns α and ω for each precursor p and each grid cell n were estimated from CTM simulation results (see Pisoni et al., 2017 for more details).

Even though the previous equations remain the same/unvaried everywhere in the whole calculation domain, the values of α and ω are grid-cell specific. The parameter α is related to the amplitude of the function and provides information about the relative importance of an emission precursor with respect to another, whereas ω provides information on the speed of decrease of the emissions impact with distance. Both α and ω are identified through a least-square estimation, using the underlying CTM simulations. This identification process also provided confidence intervals for these coefficients. More details on the procedure can be found in Clappier et al. (2015), Thunis et al. (2016), and Pisoni et al. (2017).

After *α* and *ω* have been computed, SHERPA can be used to evaluate concentration changes resulting from any emission reduction scenario, as it was done in this paper based on the traffic scenarios coming from TNET/TRANSTOOLS. In the current implementation, SHERPA is based on the CHIMERE air quality model, run with 2009 meteorology (an average meteorological year). To run the SHERPA, emissions of nitrogen oxides are used as input to model NO2 concentrations, while emissions of nitrogen oxides, volatile organic compounds, ammonia, primary PM and sulfur dioxide are used as input to model PM2.5 concentrations.

## 3 Evaluating the impact of the SUMP

### 3.1 Definition of the SUMP for the different urban areas

A Sustainable Urban Mobility Plan (SUMP) aims to improve accessibility of urban areas and provides high-quality and sustainable mobility and transport to, through and within the urban area. It targets the needs of the 'functioning city' and its hinterland rather than a municipal administrative region. The measures included in a SUMP are a set of policy intervention options grouped together in “packages” -rather than in isolation-so as to take into account potential synergies that address one or more urban policy challenges. The following typology of urban policy challenges was developed in ‘A Guide for Urban Transport Professionals’ by the CiViTAS CATALIST project, which supports dissemination and best practice transfer of the European Commission's CiViTAS initiative. The policy challenges considered here are:

• Health – How to create a healthy environment for citizens;
• Congestion – How to create an economically viable and accessible city;
• Safety and security – How to ensure a safe and secure urban environment and mobility;
• Participation – How to involve citizens and other urban mobility stakeholders;
• Strategic planning – How to achieve policy goals while ensuring that mobility needs of society and its citizens are met;
• Climate change – How to reduce climate change related emissions from urban transport to contribute to achieving local, national and global climate change goals (as an additional and underlying global challenge to be considered in urban mobility policies).

Starting from these challenges, Lopez-Ruiz et al. (2013) identified 22 groups of policy measures (without considering electro-mobility options) relevant to transport and mobility at urban level (see Table S1, supplementary information), with their share of contribution in NO_x_ emission reductions (as computed in the full set of SUMP considered in this work). In addition, seven different urban profiles for each NUTS-3 zone in the EU were defined by taking into account particular aspects of each zone in terms of density, accessibility, employment, population and commuting characteristics.

The potential impact of each group of measures was estimated through a wide literature review and included three dimensions:

- Avoid: avoid unsustainable transport practices;
- Shift: shift from unsustainable to sustainable transport modes;
- Improve: improve on current behavior in transport activities.

Each of these 3 dimension affects urban mobility in a different way, for example influencing demand, choice of mode or fuel consumption, but also differs depending on the urban context. The data from the case studies (Lopez-Ruiz et al, 2013) included more than 400 data points of combinations of SUMP measures with a measured impact expressed in terms of “Avoid- Shift- Improve” coefficients. A Random Forest regression model was built using those observations in order to correlate the three coefficients depending on each zones urban profile (population, density, network, modal split, etc.). The advantage of using Random Forests in this application was that it allowed for managing non-linear features and for handling feature interactions (Focas and Christidis, 2017). Applying the model for each FUA covered in the analysis resulted in a 642 (FUAs) x 22 (measures) x 3 (coefficients) matrix representing the net impact on emissions reduction across all modes in relative terms (as a share of estimated total urban transport emissions of each FUA). The variables that according to the model affect the total impact of the 22 FUAs the most are, in order of importance:

• Current level of emissions per capita
• Passenger car use intensity of NUTS-3 zone
• Road accessibility
• Rail accessibility
• Population density
• Total population

Apart from rail accessibility, all other five main variables have a positive correlation with the potential reduction in total emissions. This suggests that the efficacy of SUMPS is expected to be higher in more polluted, denser, car dependent and larger cities rather than - for example - smaller cities with a good public transport system. These results were used to evaluate the impact of each set of measures on emissions and concentrations, as shown in the next sections.

### 3.2 Absolute emission levels

The TRANSTOOLS/T-NET model family estimates the total emissions for each zone based on the standard following equation that encompasses the main factors that affect emissions. For each transport mode and pollutant:

(3) Emissions=Population•Activity•UseEfficiency•Fuelefficiency•Emissionfactor

With the following unit of measures

- Emissions [grams]
- Population [number of persons]
- activity [passengers per km/person]
- use efficiency [vehicles per km/passengers per km]
- fuel efficiency [litres/vehicles per km]
- emission factor [grams/litres]

The model is calibrated with data by EUROSTAT on country level totals in year 2015 for population, transport activity, use efficiency, fuel consumption and emissions. The approach allows the distribution of the totals among each country's NUTS-3 regions and FUAs. The base case results allow the SHERPA model to distinguish emission inventories between transport and non-transport sources.

The impact of the SUMP measures on NO_x_ emissions in absolute terms was estimated by applying the matrix of coefficients on the base emissions of each FUA. Given the variety of urban profiles and different challenges in each urban area, the results vary significantly among FUAs, even within the same country. At aggregate level, all 22 measures demonstrate certain effectiveness, with congestion pricing clearly appearing as the most effective (Table S1, supplementary information). Its effectiveness in all urban profiles is due to the direct impact on decreasing demand and the indirect impact of increasing occupancy rates and fuel efficiency.

**Fig. 1:**
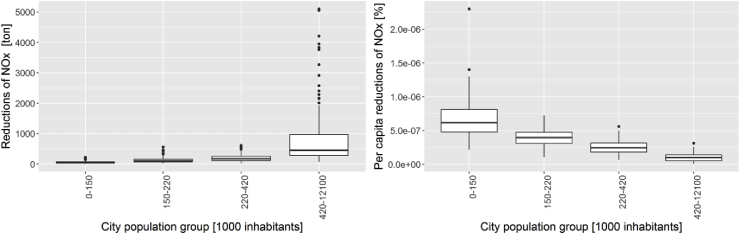
Comparison of the net emission reductions (left) and per capita emission reductions (right), grouping cities per population (note the ranges have to be multiplied to 1000, to get real population). Note that smaller cities show higher per capita emission reduction, that is to say are more efficient in implementing SUMP than big cities. Also note that city population groups are computed considering ranges of different dimensions.

The analysis performed (Fig. S1 and Fig. S2, in the “supplementary information”) allows to analysis the reductions of traffic NO_x_ emissions as a result of the application of the SUMP, for all the considered cities. The estimated emission reduction is in general moderate (on average 7%), due to the nature of the considered measures, mainly based on ‘behavioral change’. Looking at the cities grouped by population (Fig. 1), it is also clear that in terms of total emission reduction (left side) the bigger cities achieve bigger reductions; while in terms of per capita reductions (right side) it is the opposite, and the smaller cities are more efficient in reducing (per capita) NO_x_, possibly due to the easier “enforcement” of measures in comparison to bigger cities.

The results provided by T-NET/TRANSTOOLS, and shown in the previous Figures, were then transferred in a vector-based shape-file to the SHERPA model. These results were converted to the grid format used by SHERPA, and consequently, the changes in NO_x_ emissions from transport activity at urban level were input into the SHERPA model, which then computed concentration changes.

**Fig. 2:**
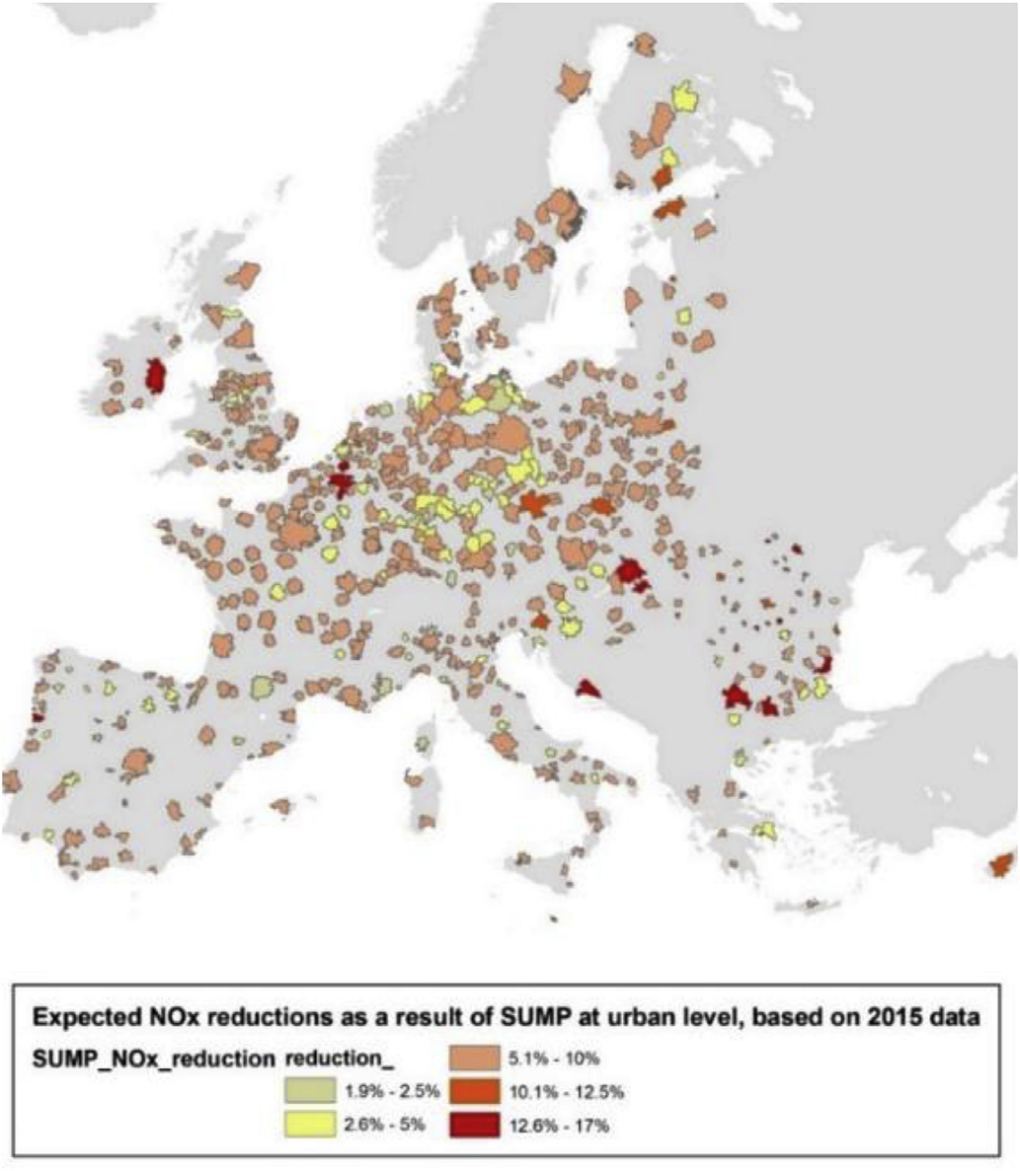
Results at FUA level across the EU, in terms of expected emission change of NO_x_.

The same analysis has been done for PM2.5, assuming that the same % reductions computed for NOx are applied to all precursors of PM2.5. This assumption (using NOx % emission reductions also for the other PM2.5 precursors) is linked to the fact that the considered measures reduce total traffic, and so act in similar way on the different precursors. As an example, Fig. 2 shows the NO_x_ percentage emission reductions for the considered cities, as resulting from the application of the SUMP measures. Emission reductions reach a maximum of 16%/17% in few cities, with average values below 10%.

### 3.3 Concentrations

As a last step, the SHERPA model has been used to simulate the concentration change due to the SUMP implementation. The SHERPA has been extensively validated in previous works; for details see Thunis et al., 2015; Clappier et al., 2015; Pisoni et al., 2017.

**Fig. 3:**
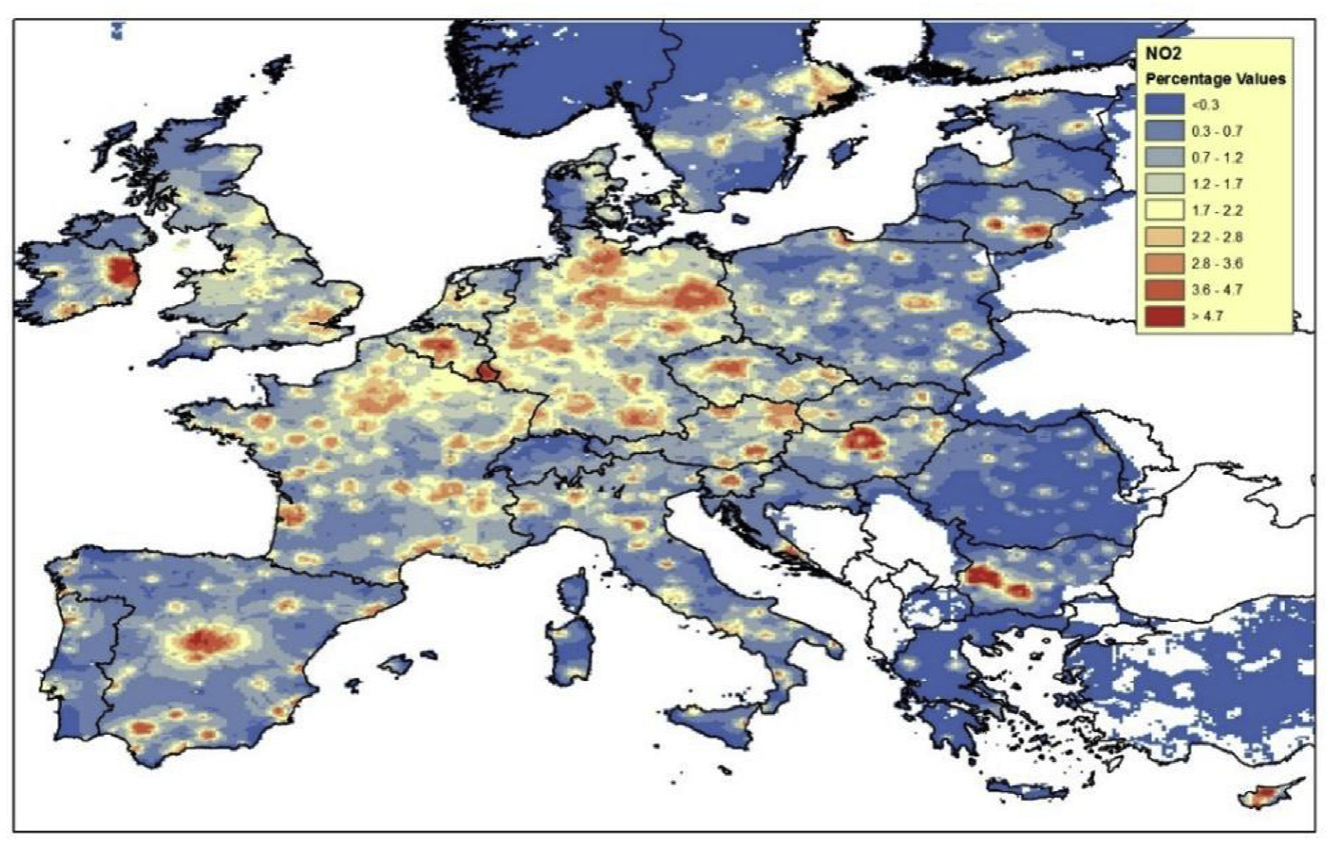
Results in terms of NO_2_% urban background concentration change, assuming the SUMP in all the considered FUAs.

**Fig. 4:**
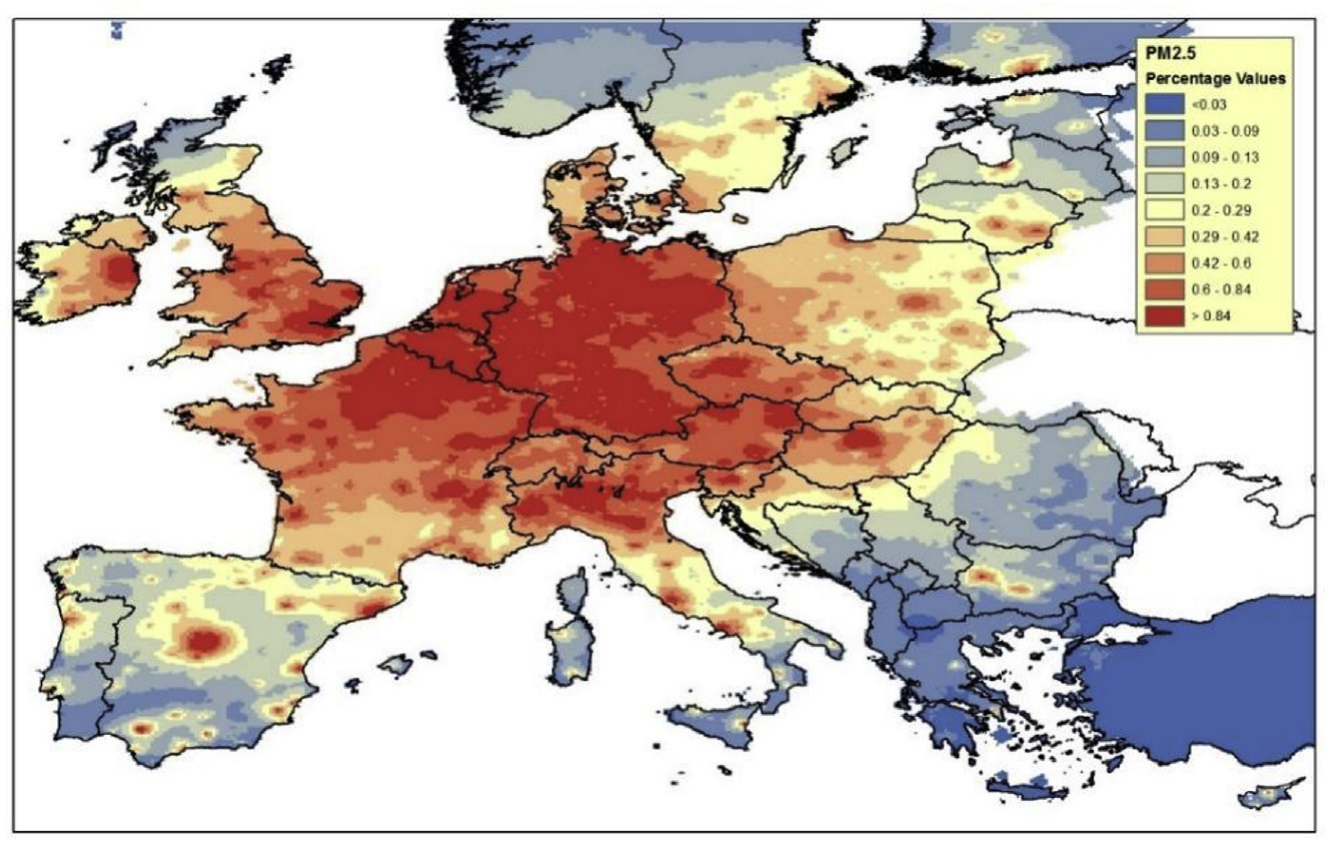
Results in terms of PM_2.5_% urban background concentration change, assuming the SUMP in all the considered FUAs.

Fig. 3, Fig. 4 show the impact of the SUMP measures, from a European perspective. This has been computed assuming that all urban areas apply the SUMP at the same time.

In particular Fig. 3 shows the impact of the implementation of SUMP in all urban areas, on NO_2_ yearly average urban background concentrations. The map shows urban background concentrations, as the SHERPA model is not able to capture the traffic station/street canyon situation (the SHERPA model works at 7 × 7 km resolution over the whole Europe).

Results for NO_2_ show that, for the considered cities, the urban background NO_2_ concentrations could be reduced of roughly 4% (see Fig. 3) due to the application of the SUMP measures. It is important to stress that this study only considers emission reductions for the traffic sector; this means that, at urban level, higher emission reductions could be achieved by acting in combination on the other sectors.

Fig. 4 shows a similar result but for PM_2.5_ yearly averages. Here, it is interesting to note that:

- The impact on local concentrations is lower than for NO_2_ because PM_2.5_ is more of secondary nature than NO_2_, and also an important fraction of the urban background pollution originates from sources located outside the city. PM2.5 therefore requires to be controlled at ‘higher level” (at regional/national/EU scales)
- The impact is less localized than for NO_2_: reductions in PM and NO_x_ emissions still have the largest effect at urban level, but they also impact surrounding areas, as PM remain for longer periods in the atmosphere than NO_x_, and impact concentrations at higher distances.

**Fig. 5:**
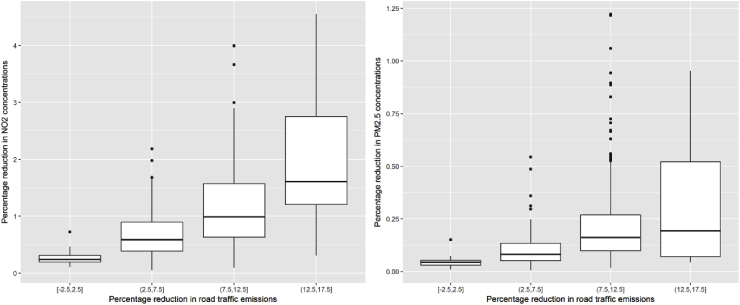
Boxplot showing how the emission change from traffic influences the NO_2_ (left) and PM_2.5_ (right) concentration change.

Fig. 5 shows a similar information than the previous map, but as boxplot. In particular, it shows how the NO_x_ and PPM traffic emission changes translate into NO_2_ (left) and PM_2.5_ (right) concentration changes. In general, changes in emissions translate in very small concentration changes. (a bit more for NO_2_, a bit less for PM_2.5_).

**Fig. 6:**
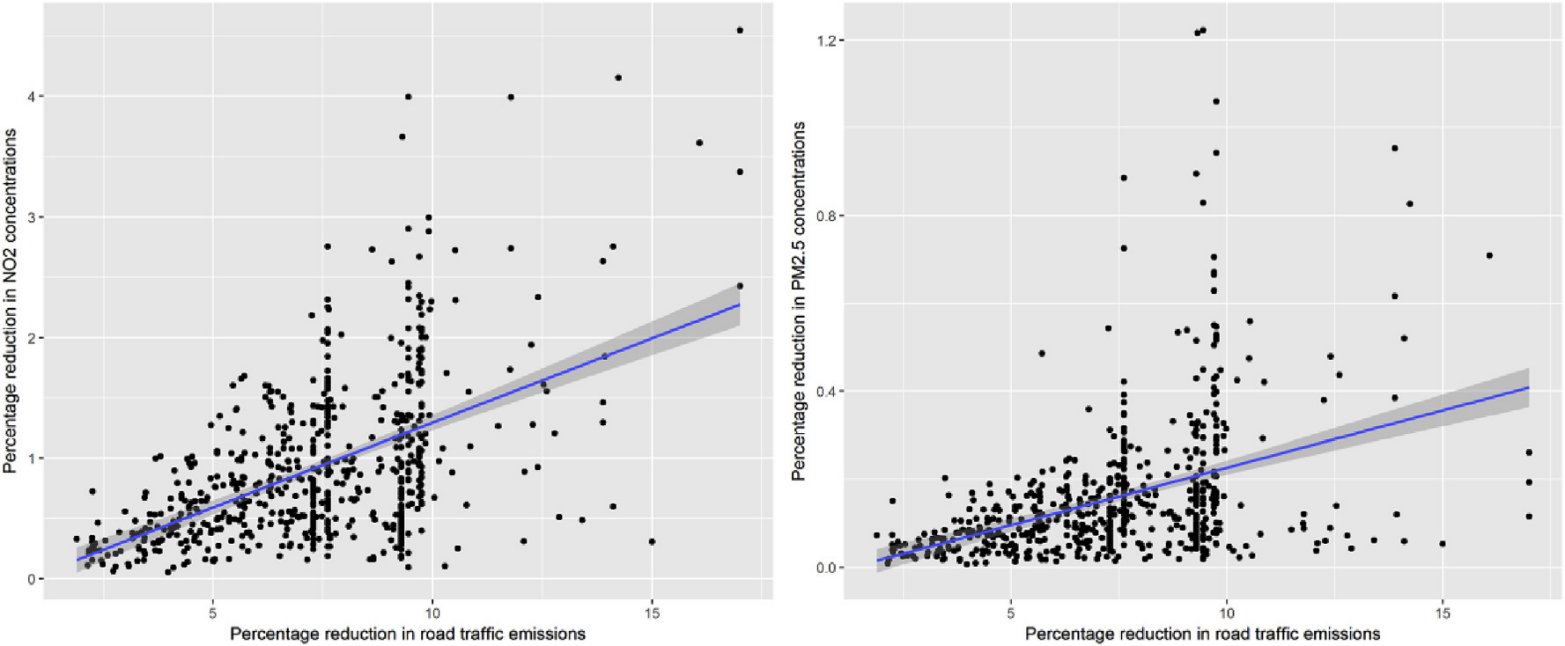
Showing that, depending on the city (each dot is a city) one can have, given the same emission reductions, very different concentration changes.

Finally, in Fig. 6 each city is considered as a dot. Given a similar emission reduction, very different concentration changes are obtained depending on the city. But even in the most ‘efficient’ case, the conversion from NO_x_ emission reductions to NO_2_ concentration change remains around 30%, while for PM_2.5_ it is around 10%.

## 4 Conclusions

Sustainable Urban Mobility Plans are an important policy instrument to improve mobility and quality of life at urban level. In this paper, the authors simulate a scenario in which 642 urban areas in Europe implement a SUMP. The results in terms of air quality improvement are reasonable - though not impressive-showing an impact on PM_2.5_ (up to 2%) and on NO_2_ urban background concentration (close to 4%).

This result should be considered as a positive outcome of SUMPs, even though the impact on air quality is only moderate at aggregate level. In fact, a number of additional considerations needs to be made before evaluating the overall impact of SUMPs at urban level:

- The SUMPs considered here do not include electro-mobility options. Considered SUMPs mainly represent "behavourial" measures, applied on a wide range of "urban profiles";
- For NO2, given an average reductions of 7% of NOx emissions, the air quality improvement on urban background concentrations is around 4%. This impact (quite limited, both in terms of emissions and concentrations) would be higher if considering not the urban background, but the impact at city/street level.
- For PM2.5, the impact is even lower (up to 2%) but again this is due the low emission reductions foreseen by the considered SUMPs. This result is in line with a previous work (Thunis et al., 2018) where the contribution of PM2.5 concentrations due to transport was quantified, for different cities in Europe. In Thunis et al., 2018 it was shown that, with reductions of 100% of the transport emissions in cities, urban background concentrations would reduce on average of 14% (even if this value would change depending on the city). The same ratio between transport emission reductions and resulting PM2.5 concentration change is also found here.
- The spatial and temporal resolution should be taken into account in the evaluation of the impacts. As in any urban level analysis, the definition of the urban area limits has a direct impact on the share of emissions from transport. As a rule of thumb, the larger the area considered as urban, the lower the density of the transport network and the lower the relative importance of transport emissions compared to those from other sources. On the temporal side, shorter analysis periods would result in higher importance of transport emissions. Functional Urban Areas (FUA) consist of urban zones with their commuting areas and as a result have a lower population and network density than their main urban centres. If analysed at peak hour level for the part of the urban area with highest road density, the impacts of SUMPs would appear as significantly higher;
- The concentrations at a traffic location (computed considering as meteorological year the 2009) can be 60% higher than urban background concentrations (as shown in Kiesewetter et al., 2013); this means that the impact, in specific traffic sites, can be more than what estimated in this work;
- This paper addresses the SUMP impact purely in terms of urban background concentrations of PM_2.5_ and NO_2_. But in reality their impact is on more ‘dimensions’, contributing to better quality of life, reduced noise, safer cities, etc. The reductions in concentrations of PM_2.5_ and NO_2_ are highly correlated with reductions in fuel consumption, CO2 emissions, congestion, noise and accident rates;
- The SUMPs correspond to actions that can be taken at local level only and therefore can influence only a subset of the factors that affect emissions from urban transport mobility. Additional measures taken at national or European level in order to influence transport demand, vehicle technology, fuel efficiency and fuel quality could certainly complement the SUMPs.
- The two considered models (T-NET/TRANSTOOLS and SHERPA) have inherent uncertainties and limitations (see i.e. Pisoni et al., 2017). However, the approach is still useful in order to reach an indicative estimate of the potential extent of the SUMP impacts and a comparison of the effectiveness between different measures and among different urban profiles.

In any case, even if the aforementioned caveats, we think this work can contribute, with its harmonized view at city level from a European perspective, to foster the discussion on new mobility packages and new mobility strategies, which are still needed at city level for much of Europe.

## Disclaimer

The views expressed are purely those of the authors and may not in any circumstances be regarded as stating an official position of the European Commission.

## Footnotes

1 A NUTS 3 zone is an administrative unit corresponding to province level under the European Nomenclature of territorial units for statistics: http://ec.europa.eu/eurostat/web/nuts.

## Footnotes

2 http://ec.europa.eu/eurostat/statistics-explained/index.php?title=European_cities_%E2%80%93_the_EU-OECD_functional_urban_area_definition&oldid=262978.
