# Supplementary material for: Evaluating the impact of “Sustainable Urban Mobility Plans” on urban background air quality
Source: J Environ Manage. 2019 Feb 1;231:249–55. doi: 10.1016/j.jenvman.2018.10.039 (PMC6331659; doi:10.1016/j.jenvman.2018.10.039)
Supplement: Multimedia component 1 [file mmc1.docx]

# SUPPLEMENTARY INFORMATION

Table S1 show 22 policy measures relevant to transport and mobility at urban level, with their share of contribution in NO_x_ emission reductions (as computed in the full set of measures considered in this work).

Table S1: SUMP policy measures and average reduction of urban transport NOx emissions per individual measure

| **Measure** | **average reduction per measure** |
| --- | --- |
| Congestion charging zones (area and cordon charging) | 0.72% |
| ICT solutions | 0.49% |
| Reallocation of road space to other modes of transport, e.g. dedicated bus lanes | 0.47% |
| Improvement of the efficiency of city logistics by the use of ICT | 0.46% |
| Public transport coverage (line and stop density, walking distances between stops) & frequencies. | 0.44% |
| Multimodal travel information provision | 0.41% |
| Low emission zones | 0.41% |
| Dedicated walking and cycling infrastructure investment and maintenance & Bike sharing schemes | 0.38% |
| Parking management | 0.38% |
| Investment and maintenance, including safety, security and accessibility | 0.34% |
| Corporate, school and personalized mobility plans (or workplace travel plans) | 0.33% |
| Information and marketing campaigns | 0.30% |
| Measures to improve the energy efficiency and environmental performance of vehicles | 0.29% |
| Taxi services (individual and collective) | 0.28% |
| Park and Ride areas | 0.25% |
| Low speed zones | 0.23% |
| Interoperable ticketing and payment systems | 0.23% |
| Car sharing & carpooling schemes. | 0.21% |
| Dynamic traffic management measures | 0.20% |
| Multimodal connection platforms | 0.15% |
| Travel information provision systems | 0.12% |
| Promotion of eco-driving | 0.07% |

Figure S1 shows a histogram of reductions of traffic NO_x_ emissions as a result of the application of the considered measures, for all the considered cities. It is possible to see how, in general, in the majority of cities considered, the emission reduction is below 10%; only a small number of cities is able to reach reductions between 10 and 15%. This low impact of the measures is also due to the fact that a number of measures proposed fall under the category “behavioural change” or “non-technical measures”, meaning that they are measures that are not (congestion charging apart) prohibiting the use of cars, but rather trying to “convince” or persuade people to avoid polluting transport options and to move to cleaner alternatives.


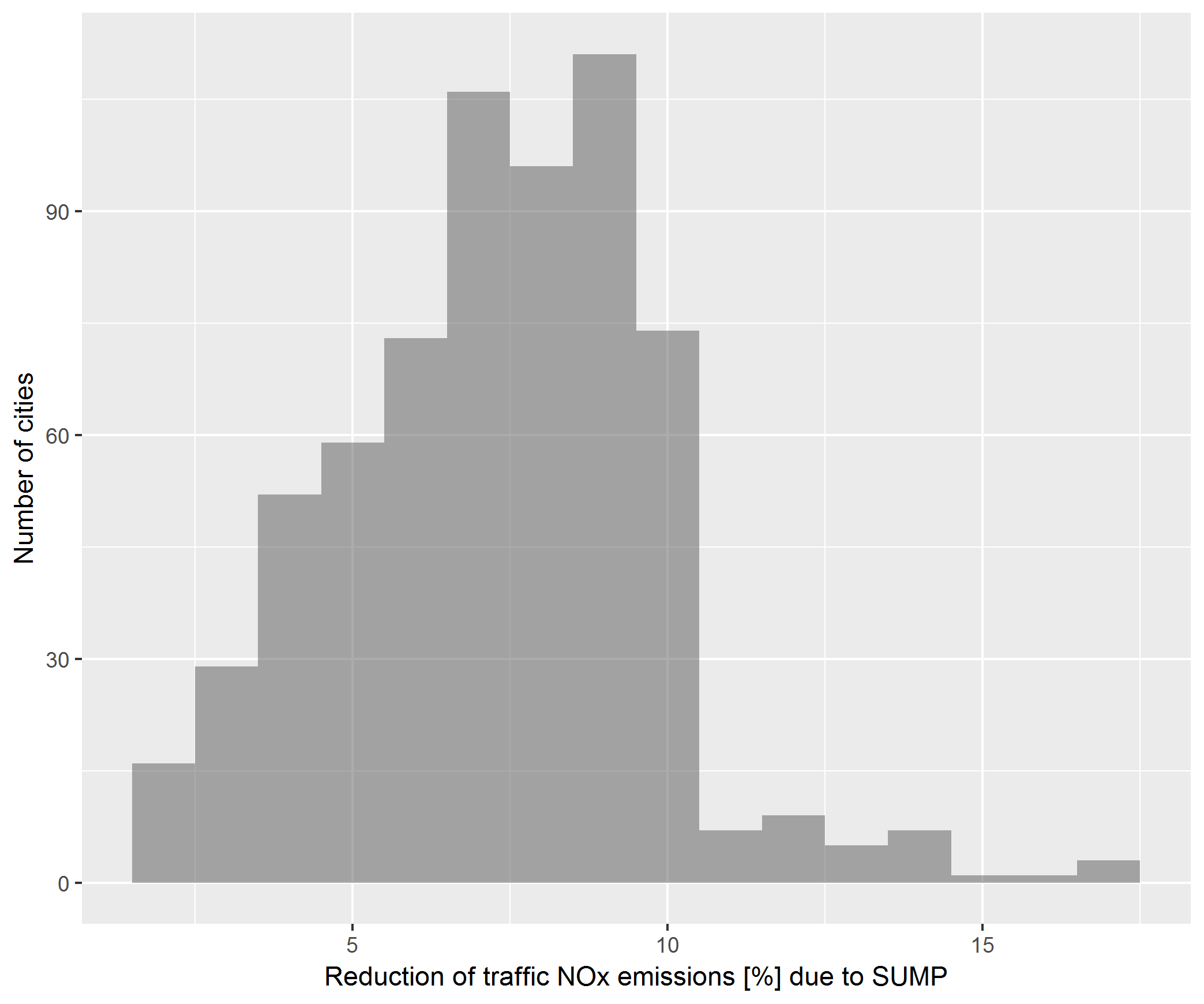


Figure S1: overview of the reductions of traffic NOx emission thanks to the SUMP, for all the considered cities.

A similar result (but in absolute values) is shown in Figure S2, in which it is clear how only few cities have a significant impact in terms of NO_x_ emission reductions, with a large “right tail” of smaller impact cities.


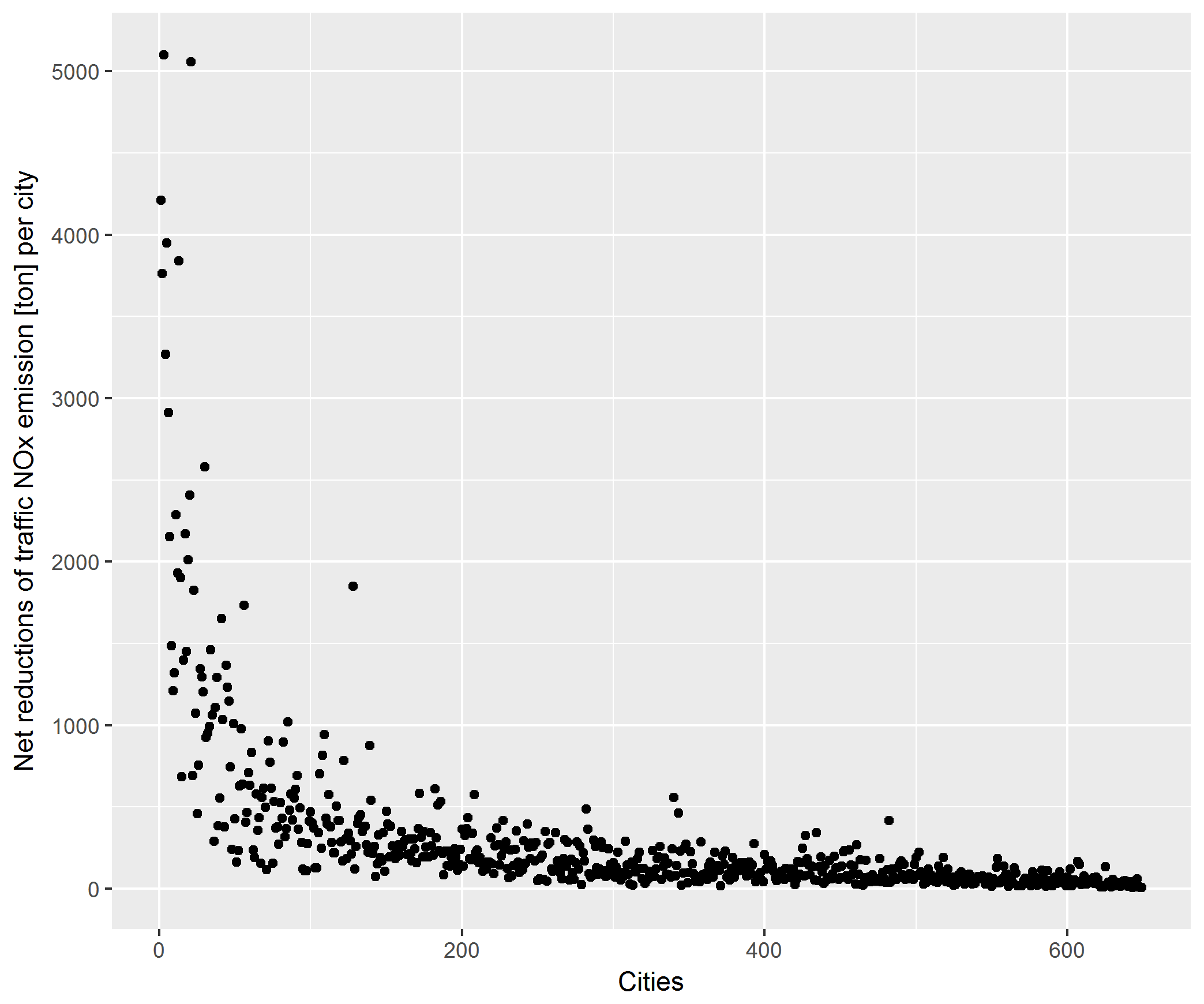


Figure S2: Net emission reductions of the SUMP, for the considered cities. Note that for a lot of cities the impact is not very strong (right tail of the curve)

Figure S3 shows the impact of the considered measures on the different member states (plus the EU-28 average) in terms of the 3 considered dimensions (Avoid, Shift, Improve).


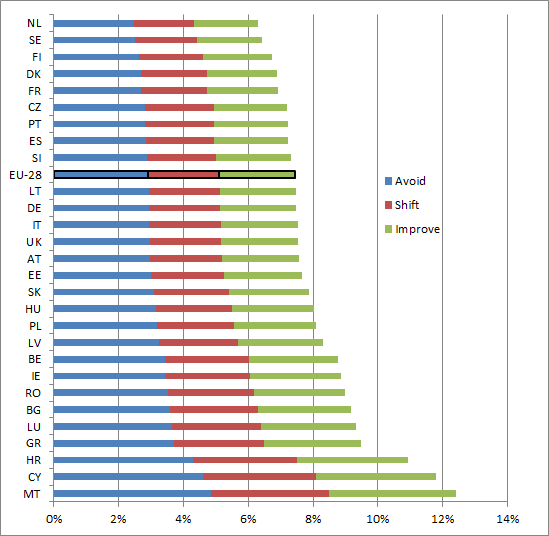


Figure S3: impact of the considered measures on the EU28 countries, splitting the contribution considering the 3 ‘dimensions’ of “Avoid”, “Shift”, “Improve” mentioned in the text.
